# The Pattern and Local Push Factors of Rural Depopulation in Less-Developed Areas: A Case Study in the Mountains of North Hebei Province, China

**DOI:** 10.3390/ijerph19105909

**Published:** 2022-05-12

**Authors:** Zhonglei Yu, Hua Zhang, Piling Sun, Yandi Guo

**Affiliations:** 1 Key Research Institute of Yellow River Civilization and Sustainable Development, Collaborative Innovation Center on Yellow River Civilization Jointly Built by Henan Province and Ministry of Education, Henan University, Kaifeng 475001, China; yzlei87@163.com (Z.Y.); guo999lucky@163.com (Y.G.); 2 Faculty of Geographical Science, Beijing Normal University, Beijing 100875, China; 3 School of Geography and Tourism, Qufu Normal University, Rizhao 276826, China; sapphire816@163.com

**Keywords:** rural depopulation, rural out-migration, local push factors, GeoDetector, mountains of north Hebei province, China

## Abstract

Rural depopulation is the most significant geographical phenomenon in rural areas during the process of urbanization. Although many studies have investigated the driving force of rural depopulation based on rural-urban migration at the macro level, the local factors, and their impact on rural depopulation from the rural areas have been not fully revealed. This paper selected the northern mountains of China’s Hebei province as a study area to explore the pattern and local push factors of rural depopulation at the rural-township levels based on GeoDetector. The main findings are summarized as follows. (1) Rural depopulation varies substantially, demonstrates spatial correlation, and is distributed in clusters. From a dynamic perspective, compare that in years 2000–2010, the population growth areas during 2010–2017 have been significantly expanded, while the sharp depopulation areas and severe depopulation areas experienced shrinkage in our study area. (2) The pattern of rural depopulation is in accordance with terrain. Rural depopulation tends to be stronger in plateaus and mountains, while relatively milder in intermontane basins, hills, and piedmont plains. (3) The agricultural suitability of natural environmental and rural economic opportunities together with climate changes were the most important driving forces of rural depopulation at local levels. Location, sparse population, and inadequate public services also contributed to rural depopulation. However, the dominant driving factors are different in the different periods. Rural depopulation was mainly driven by arable land per capita and natural environmental variables in the years 2000–2010, while the population density, location, and off-farm economic opportunities played a decisive role in the years 2010–2017. (4) Rural depopulation is a complex, multi-dimensional process driven by a combination of multiple factors including different environmental factors, economic opportunities, and location. This paper reveals the push factors of rural depopulation in underdeveloped mountainous areas by a quantitative empirical approach, inspiring increased attention to the impacts of local factors and spatial correlations on rural depopulation, and has many implications for the policy design of China’s rural revitalization.

## 1. Introduction

Rural depopulation which occurs accompanying urbanization has almost become a universal global phenomenon, and it is closely related to the sustainable development of rural areas [1,2,3]. As early as the late 19th century, rural population decline first occurred in parts of Europe and then swept across almost all of Europe and developed counties of other regions in slightly more than 100 years (between the 19th and late 20th centuries) [4,5,6], such as Canada, Australia, United States, and Japan [7,8,9,10]. In the latter half of the 20th century, many developing countries in Latin America and Asia, including China, also began to experience rural depopulation [2,11,12,13]. This phenomenon usually resulted in remarkable rural hollowing, industrial recessions, farmland abandonment, and ecological risks [14,15,16,17,18]. It has been listed as a widespread “endemic disease” by the Council of Europe and viewed as a negative process requiring remedial action [5,19]. A better understanding of the pattern and process of rural depopulation is necessary to reverse the rural decline and to seek a successful policy for sustainable development [19]. However, previous studies about population change and migration remain urban-focused and growth-focused [20,21].

The traditional explanations of rural population decline often emphasized the role of external forces at the macro level. First and foremost, industrialization and urbanization have led to many nonagricultural employment demands and higher wages, which has attracted rural migrants to urban settings [22,23]. Moreover, the trend of rural depopulation was often difficult to reverse due to the attraction of higher wages in urban areas [24]. Second, the export of international labor tends to encourage many rural laborers to transfer to foreign countries, further driving rural depopulation. For example, following the end of World War II, in the Caribbean West Indies, a large proportion of the population traveled to the United States and Britain to serve as laborers, resulting in a significant reduction in the rural population [25]. Third, the advances in agricultural production technology have increased the efficiency of agricultural production, which has freed some of the labor force from agriculture and opened the possibility of rural depopulation [7,26]. It was found to be particularly evident in the United States and central Australia, where large-scale operations and mechanized production processes are practiced [26,27]. All these forces reveal the external power of the overall decrease trend of the rural population. However, these findings do not explain why rural populations have drastically declined in some areas, while they have remained stable or even increased in other areas under the overall context of rural depopulation [23]. Therefore, further exploration of the local influential factors of rural depopulation will hopefully explain these trends and deepen the understanding of rural depopulation.

The factors related to rural depopulation at the local level emphasized by previous studies involve the natural environments, locations, and economic and social dimensions. First, many cases have shown that rural depopulation often tends to occur in mountainous and arid regions. For example, in Japan, villages in high-altitude forested areas have suffered the most severe depopulation [10]. Nepal [13], Aragones in Spain [28], and the European Alps [29] have been found to have the most severe rural depopulation in the region. In addition, environmental changes are important driving forces of depopulation [30], such as drying and heating of climate [27], drought and rainfall variability, and so on [31,32], particularly in relatively poor rural areas with a high dependency on agriculture [33]. Second, the lack of economic opportunities, including agricultural and nonfarm opportunities in rural areas has led to decreases in rural populations [34]. It may also potentially lead to increased unemployment and poverty in rural areas, which tends to further aggravate rural depopulation [12,23]. Race et al. [27] observed that rural population losses in Europe and North America were not the result of the marginalization of farmland but were instead because agricultural enterprises had ceased production. In the 1980s, the Midwestern United States experienced a dramatic decline in agriculture, which resulted in a rapid population decline in the agricultural counties, and many small towns were forced into a “struggling” condition [9]. In the 1990s, rural population losses occurred mainly in areas where nonfarming jobs were scarce [35]. Third, in terms of location, McGranahan et al. [35,36] revealed that although depopulation had mainly occurred in agricultural counties in the Great Plains of central United States, agriculture was not a trigger for rural depopulation. The main reason was that these counties were geographically remote and far from metropolitan areas. The studies from Europe also emphasized that the distance to a city and accessibility mattered considerably [19,37]. Finally, social demographic factors have also been found to play important roles in rural depopulation. Factors such as population size, structure, and density have been observed to have important effects on population migration [38]. Previous studies have shown that rural depopulation is more likely to occur in sparsely populated areas. For example, in the 1990s, most of the sparsely populated nonmetropolitan counties in the Great Plains region of Central United States experienced severe declines in their populations [35,36]. Previous studies have also found that young people often leave the countryside to receive high-level education and other services in large cities, in response to the lack of educational facilities and inadequate housing and transportation conditions in some rural areas [32,39].

Although the studies have enlightened us considerably about factors associated with rural depopulation, further studies are still required to deepen our understanding of rural depopulation at the local levels. First, the current knowledge regarding local drivers of rural depopulation is a little fragmented. Finding more quantitative and accurate scientific evidence would be helpful to better understand the causes of rural depopulation. Second, the loss of the rural population is a complex process resulting from multiple factors interacting [38]. However, few studies have explored the combined effect of diverse forces on rural depopulation. To achieve a better and clearer understanding of rural depopulation, we should not only emphasize the direct impacts of various factors on rural depopulation but also focus on the interaction influence of various factors. Third, previous studies often investigated rural depopulation at the national or macro-regional level [2,12,35]. These often failed to capture the spatial differences and the reasons for rural depopulation on a more microscopic scale. We need to get a more elaborate explanation of the rural population dynamic at the micro-level. Fourth, few studies have assessed whether the drivers of population decline differ at different stages.

China is a developing country with great agriculture and vast rural area, and the world’s most populous nation experiencing a dramatic rural depopulation. Considering the development stage and its different cultural, institutional, and cultural backgrounds, China is expected to provide excellent empirical evidence for understanding rural depopulation. In recent decades (1978–2018), the rural population declined from 0.79 billion to 0.56 billion [40]. However, most previous studies were mainly concerned with the consequences of rural population loss, such as farmland abandonment [16,41] and hollow villages [14,42]. Only several studies explored the pattern and driving factors of rural population change [2,12,43,44]. Investigating the influential factors of rural depopulation not only enlightens us regarding China’s ongoing rural revitalization strategy but also adds new accurate empirical evidence to understand the process of rural decline. 

As documented in previous literature [13], the processes of rural depopulation are particularly evident in less developed mountainous areas. For China, it is no exception. From current studies [2,45,46], we could find that the counties in undeveloped mountainous areas of China often suffered from a more serious population loss. Hence, this study chooses typical depopulation areas of China, i.e., the northern mountains of China’s Hebei province that experienced a remarkable rural population loss [47] as a case study, and utilizes a spatial quantitative method to identify the spatial characteristics and local influential factors of rural population decrease. Our aim is to reveal the local driving forces and spatial patterns of rural depopulation. The novelty of this study lies in revealing the interaction process of multiple factors behind the rural depopulation, and the dynamics of push factors of rural depopulation in different periods based on a novel spatial statistical method.

In the second section, the paper introduces the study area, along with the framework of explanatory variables, data sources, and methods. The third section introduces the results of spatial patterns and local associated variables of rural depopulation. The fourth section explains and discusses the drivers. The final section details the conclusions and implications.

## 2. Materials and Methods

### 2.1. Study Area

**Figure 1:**
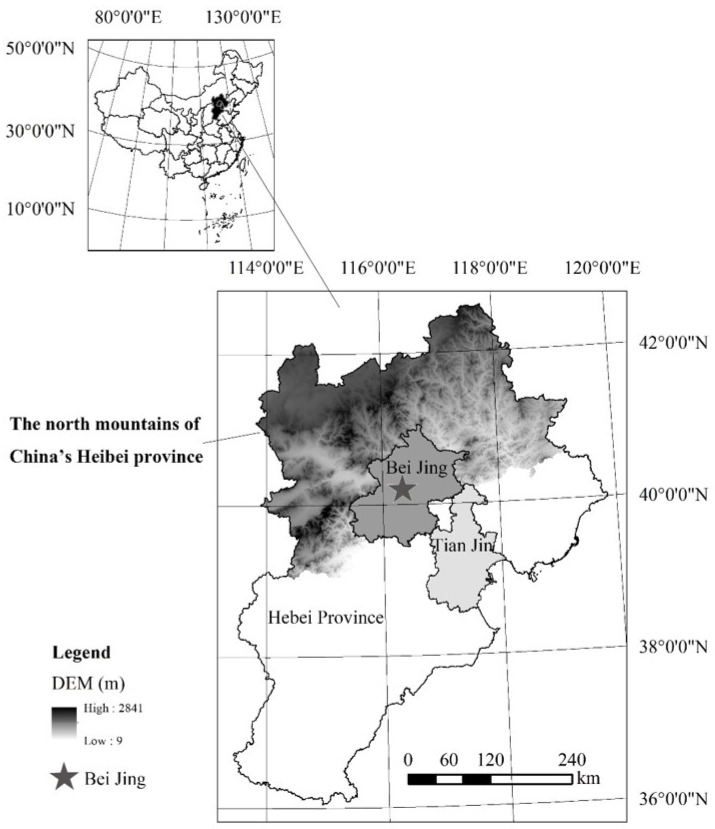
Location of the northern mountains of China’s Hebei province.

The northern mountains of China’s Hebei province were selected as the case study area because it has less development, remarkable rural depopulation, and diverse microtopography. The area was also regarded as the poverty belt around Beijing-Tianjin Metropolis by the Asian Development Bank in 2005 (Figure 1), covering the prefecture area of Zhangjiakou and Chengde, as well as the three counties of Laishui, Laiyuan, and Yi in Baoding (Figure 1) [48]. It is in the transition zone of the North China Plain and the Inner Mongolia Plateau and covers the Trail of Taihang Mountain and Yan Mountain. The territory is characterized by a large overall topographic relief and rich in geomorphic types, including hills, mountains, plains, and basins. In terms of climate characteristics, it is located in a transitional zone between the temperate monsoon climate and the temperate continental climate as well as between the warm temperate zone and the temperate zone. Accordingly, it is also an important part of the agro-pastoral transition zones of Northern China and faces strong ecosystem vulnerability and sensitivity. However, the study area plays a key role in water conservation and ecological security for Beijing-Tianjin Metropolis. Hence, strict environmental protection policies have been implemented in this area in the last decades. Under the restriction of environmental policies and the attraction of the metropolis, many economic factors preferentially flow to the more developed Beijing-Tianjin region. Consequently, the urbanization and industrialization development in the study area were observed to lag far behind that of the Beijing-Tianjin metropolitan area [49]. In addition, during the last two decades, the increasingly arid and frequent meteorological disasters have further deteriorated the rural socioeconomic situation. All of these factors have made the area one of China’s 14 contiguous poverty-stricken areas. In addition, the northern mountains of Hebei province have experienced dramatic rural population loss and it has become one of the most typical population net outflow areas in Northern China [47]. Therefore, this region represented a typical case study area for rural depopulation.

Considering that rural townships have the most detailed spatial scale in the census data of China, in this research, the rural townships were taken as the basic analysis objects. Since the population changes were greatly influenced by the administration practices, the state-owned farms and forested areas were not included in the scope of analysis. In addition, the urban areas and the capital towns (Chinese “Chengguan zhen”) of counties that are often considered small cities were also excluded from this study. We obtained 442 rural townships to examine the rural population depopulation in the north mountains of Hebei province. 

### 2.2. Measure of Rural Depopulation

According to current literature [4], when the number of people living in a rural area has decreased compared with the previous census, we can define that as rural depopulation. Considering that the large-scale rural out-migration and rural decrease in this area mainly occurred in the 21st century, we focused on the depopulation process from 2000 to now based on China’s fifth and sixth census data (in the years 2000 and 2010, respectively). As the data of the Chinese seventh census in 2020 at the rural-township level has not been publicly released completely, we chose the latest statistics of the rural resident population, which were sourced from the China County Statistics (Township Volume) in 2017, to replace the seventh census data. The rural depopulation was measured by the rate of rural population decline, i.e., the proportion of the reduced population between 2000 and 2010 to the resident population in 2000, and the proportion of the reduced population between 2010 and 2017 to the resident population in 2017. Then, we used descriptive statistical methods to analyze the characteristics of rural depopulation. The statistics used included the mean, variance, maximum, and minimum.

### 2.3. Framework of Potential Influential Factors

**Figure 2:**
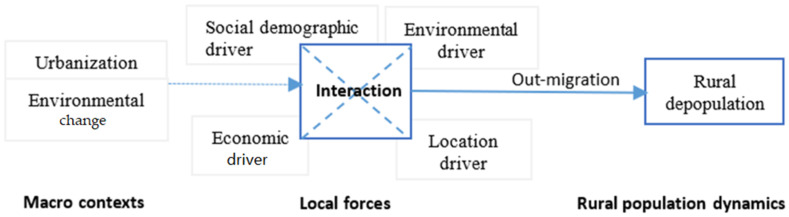
An analysis framework of rural depopulation.

Rural out-migration is a fundamental driver of rural depopulation [50]. Therefore, we can understand rural depopulation by exploring the local push drivers of rural out-migration. In recent literature, migration is considered as an adaptation to environmental changes motivated by social, environmental, economic, demographic, and political drivers, as well as environmental change through interlinking with the mentioned drivers [38,51,52]. Inspired by this framework and previous studies, we understand rural out-migration in a context of urbanization and environmental change in this study area, as an adaptation to urbanization and environmental change. The local forces of rural depopulation mainly resulting from out-migration should include environmental, economic, location, and social demographic drivers, as well as the interaction between different drivers (Figure 2). According to the framework, we selected the potentially influential factors of depopulation based on the specific conditions of the study area at the rural township level. 

#### 2.3.1. Natural Environmental Factors

The existing studies revealed that environmental changes and the local natural environment had significant impacts on the loss of rural population [53]. However, specific factors have been found to vary in different areas, such as the dry and hot climate in central Australia, and drought in Ethiopia [27,32]. The northern mountains of Hebei province are in a transitional zone between semi-arid and semi-humid climatic zones, where the climate changes which threaten local rural livelihoods are remarkable. Therefore, the rainfall variation as the characteristic of environmental change, the annual rainfall, and the annual temperature representing climate conditions associated with agriculture were included in the scope of the investigation. Then, by considering the diversity of the geomorphology associated with rural development, the topography type as the indicator of terrain was taken into consideration.

#### 2.3.2. Economic Factors

The rural areas with low income and inadequate economic opportunities would suffer more remarkable out-migration and consequently tend to experience more intense rural depopulation, especially in the context of the natural population growth is very low [34]. Income levels can be represented by the per capita income of rural residents. Economic opportunities in rural areas include both agricultural and nonagricultural economic opportunities. When there are nonagricultural employment opportunities available with a satisfactory wage in local areas, based on social rationality, the labor will tend to choose local employment rather than migrate out for employment [54]. An agricultural economy includes sectors such as farming (planting), forestry, animal husbandry, and fishing. The economic potential of planting mainly depends on the area and fertility of cultivated land. We selected per capita cultivated land area and fertility of cultivated land to represent the economic potential of planting. Referring to Wang et al., the fertility of cultivated land was expressed as the average grain yield per unit area of cultivated land over several years [55]. In addition, due to the unavailability of data on the economic potential of forestry, animal husbandry, and fishery, the total agricultural density was used to represent the total agricultural economic opportunities. The off-farm economic opportunities were expressed by the gross output value of local township enterprises. The higher output values indicated that more nonagricultural employment opportunities were provided and losses of local labor had been alleviated. All these variables are shown in Table 1.

#### 2.3.3. Location Factors

Remote areas are known to be the vulnerable areas for declines in rural populations. In contrast, the areas near urban settings easily accept the good public services and economic spillover effects of urban and adjacent metropolitan areas, and, therefore the rural populations tend to increase instead of decrease. Hence, this study selected the distances of the towns from the capitals of cities and counties where they belong, to represent the extent of remoteness in the countryside (see Table 1).

#### 2.3.4. Social and Demographic Factors

Populations tend to decrease in rural areas with sparse populations and inadequate service facilities [35,39]. The current study chosed population density as a demographic factor affecting rural depopulation at a regional level. In terms of social services, it was found to be difficult to collect specific data on education, medical, and other social services at the township level in the study area. However, Carpenter et al. claimed that when the variables of the subject under investigation could not be directly observed, the measurable surrogates with causal relationships to the variables could be used to replace that [56]. Considering the local fiscal revenue determined the investment and maintenance capacity of social services and public facilities considerably, township financial revenue was chosen as an alternative indicator representing social services.

### 2.4. GeoDetector

GeoDetector is a novel spatial statistical method to detect the relationships between geographical phenomena and their potential driving factors, which has been proposed by Wang et al. and is popular in many fields in recent years [57,58,59], especially in rural studies [60,61]. The core idea of GeoDetector was that if the changes in certain environmental factors and geographical phenomena have significant spatial consistency, the environmental factors will play a decisive role in the occurrence of such geographical phenomena [57]. The decisive role can be estimated by a *q* value, which can be calculated as follows:

(1) $$q=1-\frac{\sum_{h=1}^{l}n_{h}\sigma_{h}^{2}}{n\sigma^{2}}$$

**Figure 3:**
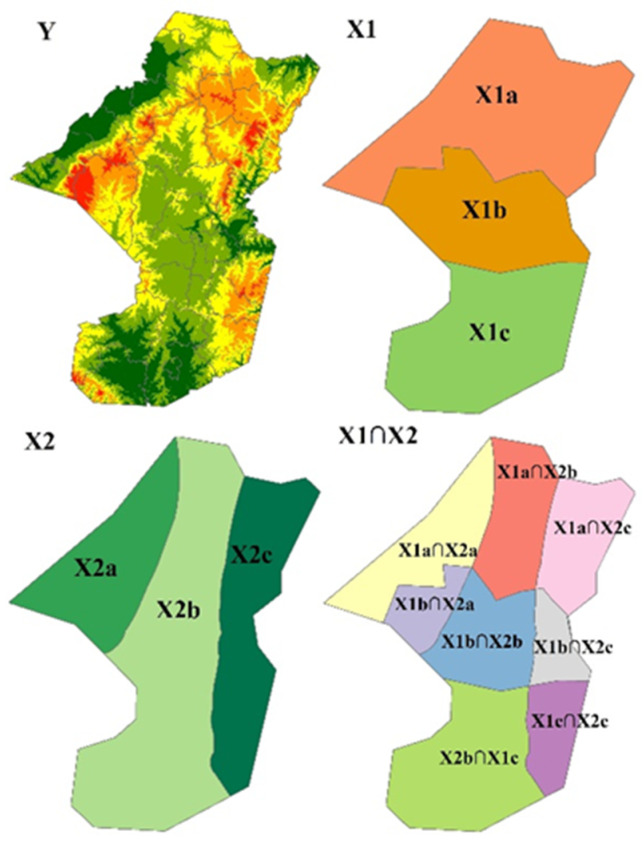
Graphical representation for interaction analysis of variables in GeoDetector. Source: drawn by the authors according to Wang and Xu (2017), online: http://www.geog.com.cn/article/2017/0375-5444/0375-5444-72-1-116.shtml (accessed on 6 May 2022).

In Formula (1), *q* represents the power of the determinant of rural depopulation, *h* ∈ [1, *l*] indicates the partition of a certain environmental factor; $n_{h}$ indicates the number of samples of this factor in the *h*-type area; *n* represents the total number of samples; $\sigma_{h}^{2}$ indicates the sample variance of the observational variables in the *h*-type area of this factor; and $\sigma^{2}$ indicates the total variance of the observational variables in all of the sample units of the study area [62]. Then, by taking the distribution of the geographic elements shown in Figure 3 as an example, factor X1 was divided into Xl_1_, Xl_2_, and Xl_3_ types, and *h* was 1, 2, and 3. Then, $\sigma_{1}^{2}$ represents the variance of the observational value of the Xl_1_ sample, and so on. If factor X1 has an impact on the observed geographical phenomena Y, the transformed *q* value should conform to the noncentral F distribution, which can then be verified through the non-center parameter λ of this distribution [62]. The calculation formula can then be written as follows:

(2) $$F=\frac{n-l}{l-1}\frac{q}{1-q}\;\sim F\left(l-1,n-l;\lambda \right)$$

(3) $$\lambda =\frac{1}{\sigma^{2}}\left[\overset{l}{\underset{\begin{array}{c} h\\ h=1 \end{array}}{\sum }}{\bar{y}}_{\begin{array}{c} h\\ h \end{array}}^{2}-\frac{1}{n}{\left(\overset{l}{\underset{\begin{array}{c} h\\ h=1 \end{array}}{\sum }}\sqrt{n_{\begin{array}{c} h\\ h \end{array}}{\bar{y}}_{\begin{array}{c} h\\ h \end{array}}}\right)}^{2}\right]$$

In the above two formulae, *y_h_* represents the average characteristic value of Y in the *h*-type area of a factor; *λ* is the non-center parameter to be verified, and; the query statistical checklist of the *λ* value can be referred to in an effort to confirm whether the *q* value is significant [62]. The value of *q* is in the range [0, 1]. However, when *q* = 0 and is statistically significant, this factor will have no impact on the observed geographical phenomenon. Additionally, when *q* = 1 and is significant, this factor plays a completely decisive role in the observed distribution of the geographical phenomenon Y.

In addition, considering that the different environmental factors may interact with the observational objects, multiple types of environmental factor areas can be spatially overlapped. For example, in Figure 3 we can detect the interaction effect of X1 and X2 on Y by calculating the *q* value in the same manner, based on the area types generated by the spatial overlapping (X1∩X2).

Compared with the regression model, Geodetector does not require observation variables to satisfy the preconditions of the same variance and normality [57]. Additionally, it can take both qualitative and quantitative variables into account, and can also identify the interactions of multiple factors on dependent variables. The purpose of this study was to identify which factors affected the rural depopulation in the study area, which involved a variety of qualitative and quantitative geographical factors as well as the interactions of different factors on the rural depopulation. Therefore, the GeoDetector displays obvious advantages in this study. In addition to terrain factors, all other variables in Table 1 were classified into five categories by a natural break in ArcGIS, and then used in GeoDetector. The GeoDetector calculation was carried out in GeoDetector software (Beijing, China) based on Excel by Professor Wang’s team at the Institute of Geographic Sciences and Natural Resources Research, Chinese Academy of Sciences (See http://www.geog.com.cn/article/2017/0375-5444/0375-5444-72-1-116.shtml (accessed on 6 May 2022) and http://www.geodetector.org/ (accessed on 12 April 2017)).

## 3. Results

### 3.1. The Pattern of Rural Depopulation in the North Mountains of Hebei Province

As shown in Table 2, there are 356 rural townships (accounting for 80.52%) in the study area that experienced depopulation from 2000 to 2010, while only 86 townships displayed slight increases in population. The average change rate of populations in the study area during the observed decade was −8.69%. The average depopulation rate ranged up to 12.83%, and the highest depopulation rate was observed up to 66.3%. The standard deviation of the population change rate was determined to be 15.5%. From 2010 to 2017, the rural population continued to decline. The average rate of rural depopulation was 14.39%, with a standard deviation of 12.71%, and the rural township with depopulation had decreased to 277 (Table 2). The results suggest that rural depopulation occurred in most of the study area, and striking disparities characterize rural population change at the rural- township level.

**Figure 4:**
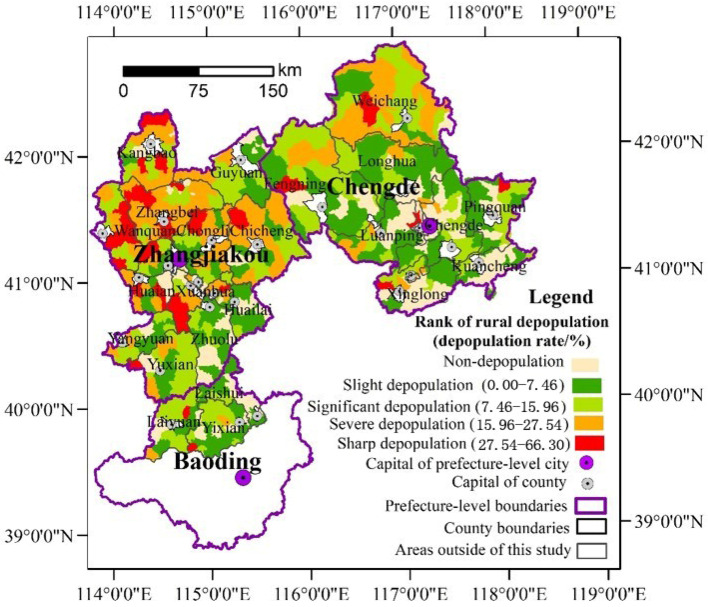
Spatial pattern of rural depopulation in the northern mountains of China’s Hebei province from 2000 to 2010.

**Figure 5:**
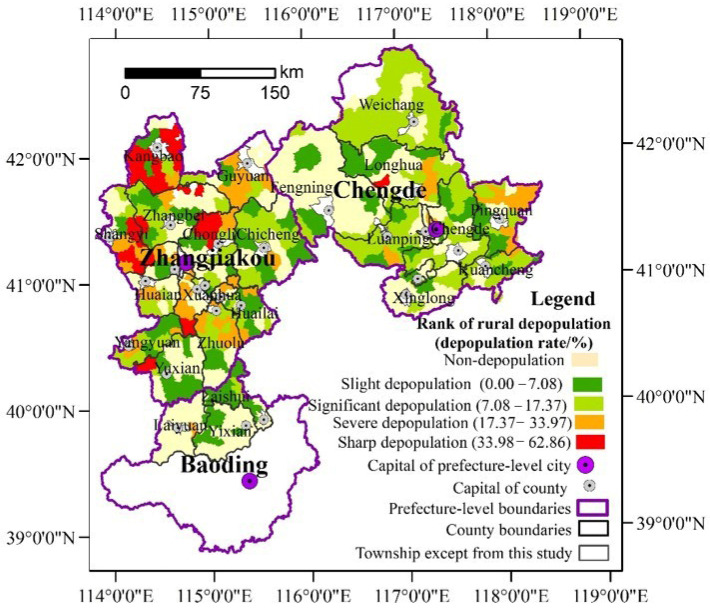
Spatial pattern of rural depopulation in the northern mountains of China’s Hebei province from 2010 to 2017.

From the spatial perspective, the rural depopulation in the study area is characterized by imbalance. With the classification method of natural breaks, the rural depopulation rates were divided into four categories as follows: sharp depopulation, severe depopulation, significant depopulation, and slight depopulation. As shown in Figure 4, from 2000 to 2010, the sharp and serious depopulation areas were mainly distributed northwest of Zhangjiakou as well as in the north of Chengde. The southern part of Zhangjiakou and the adjacent areas of Baoding were mainly observed to depopulate slightly or to increase. In comparison, from 2010 to 2017, the pattern of rural depopulation changed a little. The sharp depopulation of rural townships was mainly distributed in Kangbao and Shangyi counties in the north and west Zhangjiakou, while the severe depopulation areas were mainly concentrated in the suburbs of Zhangjiakou City and the east of Chengde (Figure 5). Overall, it was observed that the rural depopulation rate in the south of the study area was lower than that in the north, while that in the east was lower than in the west. From a dynamic perspective, the growth areas have been significantly expanded, while the sharp depopulation areas and severe depopulation areas experienced shrinkage. 

**Figure 6:**
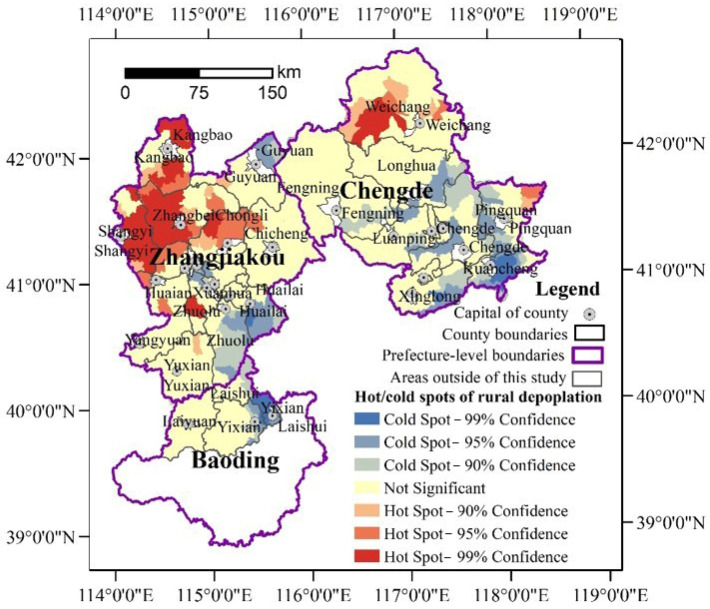
Cold and hot zones of rural depopulation in the northern mountains of China’s Hebei province from 2000 to 2010.

**Figure 7:**
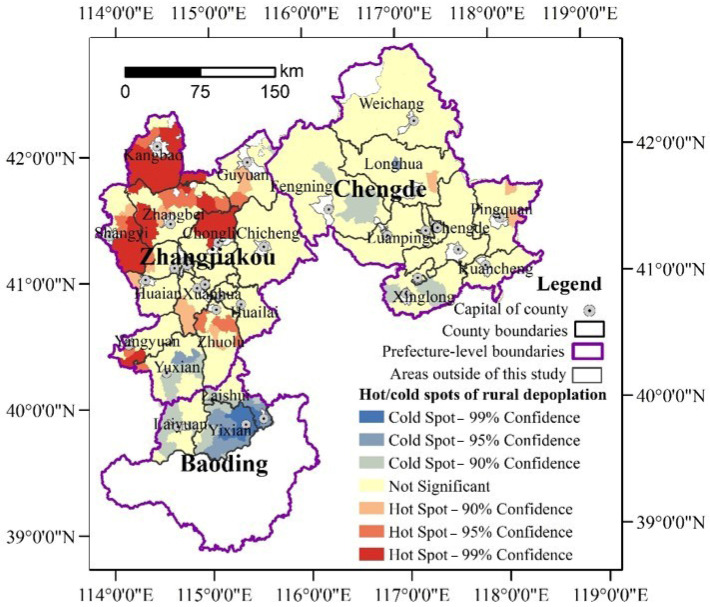
Cold and hot zones of rural depopulation in the northern mountains of China’s Hebei province from 2010 to 2017.

Moreover, rural depopulation is characterized by convergences. As Figure 4 and Figure 5 show, the rural towns with the same or similar depopulation rates tended to be concentrated. The south of Chengde and the three counties in Baoding were determined to be mainly slightly depopulated and non-depopulated towns. Additionally, the sharp and significant depopulated areas were mainly located in the northwest of Zhangjiakou. The global autocorrelation analysis further reports that the Global Moran’s index of rural depopulation rates had reached 0.3306 (*p* = 0.00) during 2000–2010 and 0.2695 during 2010–2017 (*p* = 0.00), which indicated that rural depopulation is interdependent in spatial. Additionally, the spatial hot spot detection identified significant hot and cold spots (Figure 6 and Figure 7), further clarifying the rural depopulation convergence. The hot spot zones of rural depopulation mainly formed in the west and north of Zhangjiakou, and Weichang County in north Chengde, which belong to the Bashang Plateau and Yanshan Mountains, respectively. The cold spot areas were mainly distributed in the southwest of the study area around Beijing as well as south of Chengde, and near Chengde municipal district. All these findings indicated that rural depopulation involves spatial dependence and is distributed in clusters.

**Figure 8:**
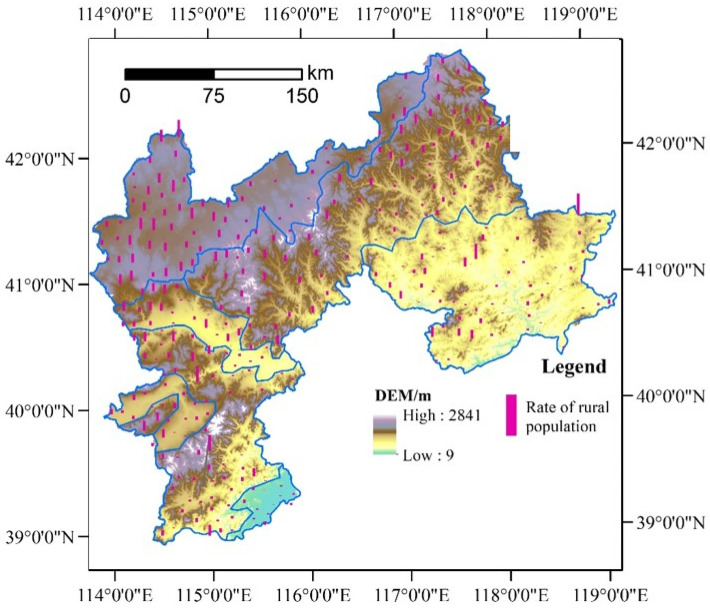
Varying rural depopulation in the northern mountains of China’s Hebei province from 2000 to 2010.

**Figure 9:**
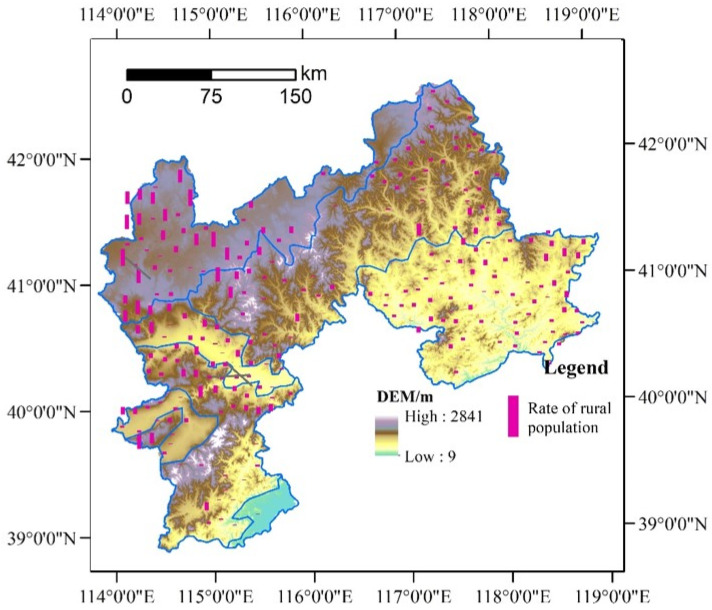
Varying rural depopulation in the northern mountains of China’s Hebei province from 2010 to 2017.

In addition, the rural population shrinkage tends to occur in plateau and mountain terrain areas, while it is less severe in the piedmont plain and hilly areas. As detailed in Figure 8 and Figure 9, the towns with a relatively high rate of depopulation were mainly located in Bashang Plateau, Taihang Mountains, and Yanshan Mountains. However, in the intermontane basin, the piedmont plain of Taihang Mountains, and the hilly area of Chengde, most rural areas have a relatively low depopulation rate. Generally, both the mountainous areas and the flat topographic areas with high altitudes suffer from a high risk of rural depopulation.

### 3.2. Results of Factor Detection in GeoDetector Model

The results of GeoDetector show that most of the selected explanatory variables impacted rural depopulation. As shown in Table 3, except for the distance to the county-level capital, all the other factors passed the significance test during the period of 2000–2010. From 2010 to 2017, all the economic factors, location factors, and social factors were statistically significant, but only the annual average temperature and the terrain affected rural depopulation.

From the aspect of natural environmental factors, from 2000 to 2010, the variables of annual average temperature and annual average rainfall obtained a coefficient of 0.2143 and 0.1334, which ranked first and third respectively. Moreover, the rainfall variability and terrain ranked fifth and seventh with the coefficients of 0.1143 and 0.0708, respectively. However, the annual average rainfall and rainfall variability were not associated with rural depopulation, while the annual average temperature and terrain obtained a coefficient of 0.1692 and 0.1009, respectively. The results suggested that the impacts of the natural environment tended to weaken. 

In regard to economic conditions, from 2000 to 2010, the arable land per capita and agricultural economic density were the most important drivers. They ranked second and fourth with the coefficients of 0.2038 and 0.1219 respectively. Then, the off-farm economic opportunity and rural residents’ income followed with coefficients of 0.0663 and 0.0451 respectively, ranking eighth and ninth. The fertility of cultivated land was also associated with rural depopulation with a coefficient of 0.0351. Compared to the period from 2000 to 2010, all the variables’ coefficients from 2010 to 2017 changed a little. The off-farm economic opportunity and agricultural economic density became the most important drivers of rural depopulation, with a coefficient of 0.2528 (ranking second) and 0.2235 (ranking third), respectively. The fertility of cultivated land and rural residents’ income also obtained higher coefficients (0.1363, 0.1206), ranking 5th and 8th respectively. The arable land per capita was still statistically significant, but with a lower coefficient of 0.1041. The rank dropped from 2nd to 9th.

Regarding the location factors, during the period from 2000 to 2010, only the distance to the prefectural capital was significantly associated with rural depopulation. It ranked 12th with a low coefficient of 0.0266. The result suggested that rural depopulation in this study area was only associated with the location relative to the prefecture-level city center, while the location relative to the county-level capital had little impact. However, from 2010 to 2017, the associated relationship between rural depopulation and the two location factors strengthened a lot. The coefficient of distance to the prefectural capital increased to 0.1266, while the distance to county-level capital had a statistically significant coefficient of 0.1238. The results meant that the location’s impact on rural depopulation became more important.

Table 3 reports that both social factors were significantly associated with rural depopulation during the two periods. The population density and provision of public services had a coefficient of 0.0837 and 0.0383, respectively, and ranked from sixth to tenth from 2000 to 2010. In the later period, population density became the most important driver for rural depopulation with a coefficient of 0.4427 (ranking first). These results indicated the social factors’ impact on rural depopulation.

### 3.3. Results of Interaction Analysis in GeoDetector

The results also showed that each factor had a significant impact on rural depopulation when combined with each other. As detailed in Table 4, the combined influence was mainly from the natural environment and economic factors, natural environment and location, social factors and natural environment, different natural environmental factors, and different economic factors. There are 38 factor pairs with interaction determinants of more than 0.2 from 2000 to 2010. The factors that played major roles in the interaction effectiveness included annual rainfall, annual temperature, arable land per capita, off-farm economic opportunities, distances to prefecture-level capitals, population densities, rainfall variability, and terrain. Among these factor pairs participating in the interaction, arable land per capita was found to have the strongest interaction with distances from the prefecture-level capitals, with a *q* value up to 0.3007. The other factor pairs with determinants ranking in the top ten were as follows: off-farm opportunities and annual rainfall (*q* = 0.2795), population densities and arable land per capita (*q* = 0.2780), arable land per capita and annual temperature (*q* = 0.2751), distances to prefectural capitals and annual temperature (*q* = 0.2733), annual temperature and annual rainfall (*q* = 0.2731), arable land per capita and terrain (*q* = 0.2702), population densities and annual rainfall (*q* = 0.2624), distances to county-level capitals and arable land per capita (*q* = 0.2610), and rural residents’ income and annual temperature (*q* = 0.2609).

From 2010 to 2017, the combined influence from factor pairs had changed a lot, with stronger and different interactions processed. By comparison, the variables playing a determining interactive role were mainly in the dimension of location and economics. The social factors, especially population density, were playing an increasing role with other factors in population decline. As shown in Table 4, there were 41 factor pairs with interaction determinants of more than 0.25. Most of them were distributed in the dimensions of economic, location, and social demographic. The factor pair of population density and off-farm economic opportunity got the maximum coefficient, which ranged up to 0.5139, followed by the coefficient of population density and fertility of cultivated land (*q* = 0.4929). The population density obtained coefficients of more than 0.4 with all the other variables. Additionally, the agricultural economic density and off-farm economic density also mattered a lot, having a coefficient of more than 0.25 with most other variables. Overall, the above results indicated that rural depopulation was driven in a combined manner by multiple factors.

## 4. Discussion

### 4.1. The Imbalance of Rural Depopulation at the Local Level

Figure 4 and Figure 5 suggest that striking disparities characterize rural depopulation at the rural township level. Although rural depopulation occurred in most of the study area, there were still some rural townships that were found to have increased. Moreover, for the process of rural depopulation, there were also remarkable spatial differences in the rate of depopulation. These findings echoed the conclusions of previous studies that the population changes in rural areas were spatially unbalanced [2,3,23]. Hence, we can believe that rural depopulation was an inevitable result of urbanization, but not all rural areas would experience depopulation. As the population is one of the key drivers of rural development [63], the rural areas with serious depopulation would suffer from rural decay, while the rural areas with population growth would have promising prospects. Therefore, the policy designers of rural revitalization should lead the population in rural areas with serious depopulation to move to the vibrant rural areas, and allocate enough resources for the population growth in those rural areas.

### 4.2. The Trigger Role of Agricultural Suitability of Natural Environmental and Climate Changes

**Figure 10:**
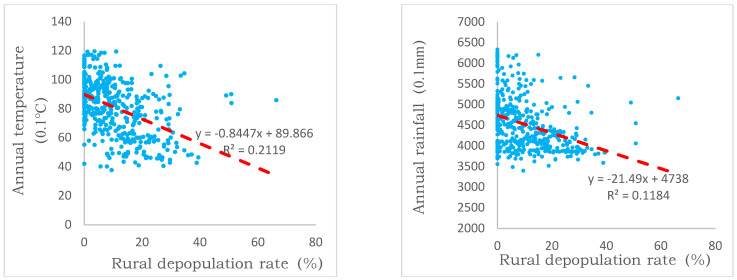
Scatter charts of rural depopulation and climate factors.

The results outlined in Section 3.2 and Section 3.3 suggest that the disparities of rural depopulation can be explained substantially by the natural environment. In terms of climatic conditions, heat and rainfall are the basic natural conditions for agricultural production, and dry and cold climates often restricted the development of agriculture and animal husbandry. The study area was located in the transition zone between the semi-arid and semi-humid regions, with significant differences in annual average temperature and annual average rainfall. Consequently, the rural population change varied a lot. As shown in Figure 10, the rate of rural depopulation tended to increase with decreases in the annual temperature and annual rainfall. This suggests that rural areas with poor rain and heat conditions would be more susceptible to serious rural depopulation.

Moreover, as the results imply, climate change proxied by the rainfall variability affected the rural depopulation markedly. During the past few years, the rainfall instability in the northern mountains of Hebei has been remarkable as climate change accelerates, while extreme weather phenomena, such as drought, floods, and hail, have frequently occurred [64]. All these greatly enhanced the instability of agricultural harvest and reduced farmers’ income. For example, in 2001, the spring rainfall in Zhangjiakou, where rural depopulation occurred seriously, was only 37.9 mm, 40% less than usual. As a result, 43.41% of cropland had not been cultivated. The rainfall from June to September was only 56.8% of the normal rainfall, which caused 146,700 hectares of crops to suffer blight [65]. In addition, in 2007, the rainfall of Zhangjiakou during the spring sowing season was only 39.4 mm, 40% less than usual. Moreover, the rainfall from June to August was only 172.5 mm, 34.7% less than usual, and especially in Bashang Plateau, the rainfall only reached half of the usual amount [66]. In this case, out-of-town employment became the farmers’ preferred choice. This observation was also found to be consistent with findings in other regions of the world [32,33,53]. For example, in Ethiopia, drought and rainfall instability has become two of the main causes of rural population losses [32].

In addition, the results show that terrain was significantly associated with rural depopulation. Our further analysis revealed that in the high-relief mountainous areas and hills at high or middle altitudes, the average rate of population decline was as high as 14.17%. It was 12.18% in the moderate-relief mountainous areas at medium altitude. Surprisingly, during 2000–2010, the average rate of rural depopulation in the medium- and low-altitude plains was also relatively high (11.66%), whereas it was the lowest (only 6.03%) in the low-altitude and low-relief mountainous areas. The rural depopulation also showed a similar pattern in the period 2010–2017. These findings indicated that although the determinant of terrain was not very high, the mountains and hills at high or medium altitude, and the plains at medium and low altitude mattered considerably for rural depopulation. The reason for this trend was that the agricultural production processes in the plain areas were mainly mechanized production, which tended to be intensive and large-scale. These production processes were prone to producing a surplus labor force, which had increased the possibility of rural depopulation, especially in the case of the absence of local employment opportunities. In mountainous areas and medium or high hills, the arable land was relatively scarce and often difficult to be farmed by mechanized operations and large-scale management models. This would lead to low productivity and less income. Therefore, many of the farmers abandoned agricultural cultivation and relocated to seek nonagricultural livelihoods, which subsequently resulted in higher rates of rural depopulation.

### 4.3. Economic Opportunities Played a Key and Enhancing Role in Rural Depopulation

Previous literature revealed that economic opportunities are the key to the decrease in rural population [2,3,6]. This was also confirmed by our study. As shown in Table 3, the economic opportunities from agriculture and off-farm industry had significant impacts on rural depopulation. 

**Figure 11:**
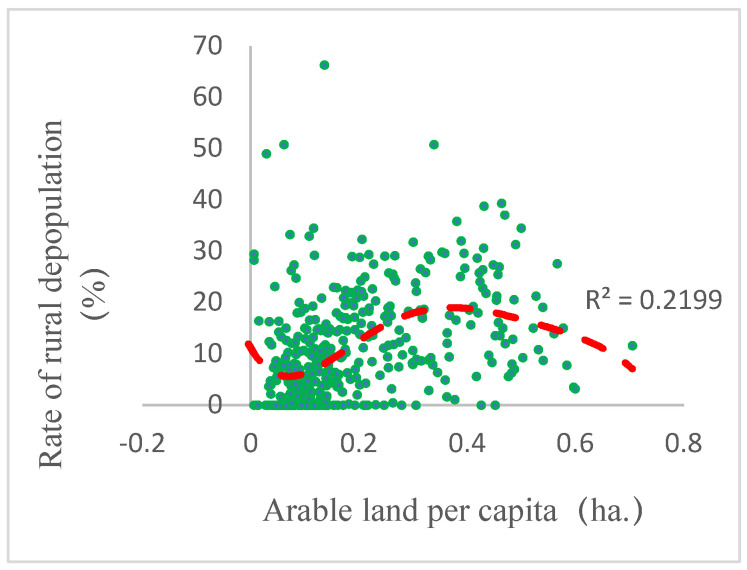
Scatter charts of rural depopulation and arable land per capita.

First, the determining force of arable land per capita ranked second from 2000 to 2010, indicating that it played a crucial role in maintaining the population. Although it ranked ninth from 2010 to 2017, the efficiency was still up to 0.1. As we all know, the small family farming system restricts the increases of labor productivity and agricultural income, and it is very difficult to provide high enough income levels to attract people [67]. This was the reason why arable land per capita affected the decrease in population. Further clarification was needed that the relationship between rural depopulation and arable land per capita was not simply positive or negative. As shown in Figure 11, with the increase of arable land per capita, the rates of rural depopulation first declined and then increased. It was in line with the trend of the rural depopulation rate with the transitions of the terrain from the mountains and hilly areas to the plain areas. This correlational relationship between the rural depopulation of arable land and terrain was not difficult to understand. In principle, the arable land per capita in hilly areas was much more than that in mountainous areas but less than that in hilly areas. In the process of urbanization, increasingly more cultivated land in the plains implemented the model of large-scale mechanized agriculture and consequently resulted in surplus agricultural labor and many out-migrants. However, in mountainous areas, the arable land was too scarce to sustain a livelihood, which also resulted in mass out-flows. Moreover, in the hill areas, the arable land was more than that in the mountains, but often could not develop the large-scale mechanized agriculture, compared to the plains. Consequently, the push for rural out-migration from per capita cultivated land is relatively less than that in mountains and plains. In addition, people living in areas of hills could often develop fruit planting, which could absorb much of the labor force and increase farmers’ income, such as in the hill areas of Chengde. Thus, the rural depopulation in hills was often less severe than in plain and mountainous areas.

Second, the fertility of arable land and the agricultural economic density affect the depopulation a lot. Regarding the fertility of arable land, in cases where the inputs of other factors for production are the same, the farmland with higher fertility will produce greater returns. The rural areas with poor land often suffer from more serious population loss, since people there are faced with less opportunity and more pressure to survive by migrating out. Analogously, the rural areas with less agricultural output per unit area also experienced a more serious loss of population. However, it was noteworthy that the determinant of the farmland productivity on rural depopulation rates was far less than the agricultural economic density (including crop and plant cultivation, forestry, animal husbandry, and fishery). This suggested that the comprehensive economic potential of agriculture had greater impacts on the rural depopulation of the study areas than the cultivated land quality, which tended to only affect the planting industry. These findings were determined to be related to the transitions of the farming areas to agro-pastoral ecotone areas, such as the counties located on the Bashang Plateau, where the output value of animal husbandry accounted for a large proportion of the agricultural activities. 

Third, the off-farm economic opportunity also mattered to rural depopulation and had a reinforcing influence. Qi et al. [68] found that job opportunities in the secondary industry and local economic level were the main drivers of rural attraction. Our result was in accordance with this finding and could suggest that the increases in off-farm economic opportunity could effectively alleviate rural depopulation to a certain extent. In particular, under the influences of people’s attachment to the countryside in China, if they were able to participate in nonagricultural employment without leaving their hometowns to obtain considerable economic returns, most rural laborers would prefer to choose local employment, especially the relatively elderly rural laborers [54]. 

Last but not the least, according to the significance of rural residents’ income and existing theory on migration, we can infer that rural depopulation occurs preferentially in poverty areas. This was consistent with the findings of previous studies [21,69]. This conclusion can also be reflected by the spatial patterns of migration and the geographical distribution of the poverty-stricken counties in China, where the concentrated poverty-stricken areas are often the areas with the most serious loss of population [45,46].

### 4.4. Sparse Population Density and Inadequate Public Services Strengthen Rural Depopulation

**Figure 12:**
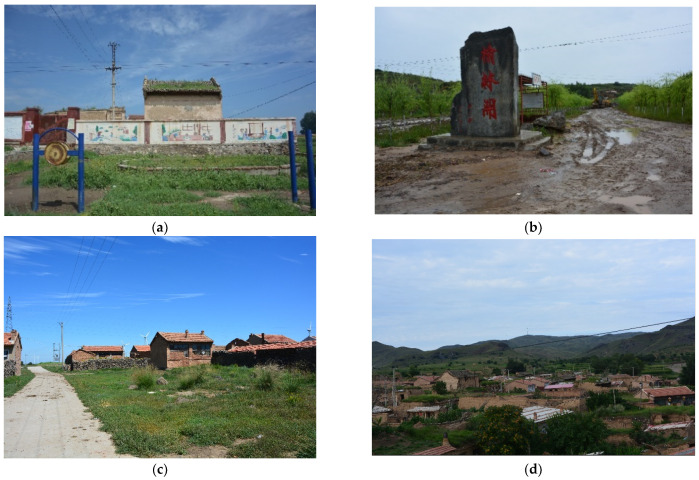
Landscapes of rural decline in the mountains of northern Hebei province, China. Note: The pictures were photographed by the authors in Yangyuan and Zhangbei of Zhangjiakou in August 2016. (**a**) Decaying public services; (**b**) Road infrastructure out of repair; (**c**) Hollowing village; (**d**) Poor living conditions.

As shown in Table 3, the population densities and social services are significantly associated with rural depopulation, and both had an increasing effect from theperiod 2000–2010 to 2010–2017. We further confirmed the findings of existing studies [35,36]. Previous studies revealed that if the population size is too small, the potential users of public services cannot maintain the operation of the service facilities [23]. As a result, those regions with a sparse population distribution have faced shortages in education, daily life, and other service facilities, which subsequently resulted in further depopulation [27,70,71]. In this study, our further analysis shows that when the population density was below 75 persons/km^2^, the rate of rural depopulation reached 12.8%. When the population density reached 500 persons/km^2^, the rural depopulation rate dropped to 3.71%. This meant that sparse population density contributed to rural depopulation. Moreover, our field investigation in 40 villages in the study area also confirmed that educational and housing facilities and transportation infrastructure were often scarcer and more run-down in small villages (Figure 12). Many villagers had therefore moved to capital towns so their children could attend school, while the villages faced decline and hollowing (Figure 9). Likewise, we can also understand the impacts of the provision of social services on rural depopulation.

### 4.5. Location Contributes to the Rural Depopulation and Has an Enhanced Effect

From Table 3, we could still believe that the decrease in rural population is associated with the location. This is consistent with the findings from Cawfey [19], Beale [26], and Mcgranahan et al. [35,36,72]. However, the impacts of location factors on the rural depopulation did not seem to be as strong as expected. As Table 3 shows, the distance to county-level capital was only statistically significant with a low coefficient of 0.1238 (ranked 7th) in the period 2010–2017. The distance to the prefecture capital was also statistically significant with a coefficient of 0.0266 (ranked 12th) from 2000–2010, and 0.1266 (ranked 6th) from 2010–2017. This may have been because the location’s driving effects were also dependent on the size and economic development of cities. In the northern mountains of Hebei province, the pull of the prefecture-level capitals was not strong enough to attract many rural migrants, due to being undeveloped economically. For the county-level capital, the capital towns of most counties in this study area were relatively underdeveloped economically and cannot provide excellent education services, and consequently lacked sufficient appeal for migrants. People often migrate to other areas with rich jobs and excellent educational services, rather than the capital of their own counties.

### 4.6. Rural Depopulation Results from the Interactions between Various Factors

As shown in Table 4, the rural depopulation was also under the integrated influence of different factors, especially annual rainfall, annual temperatures, rainfall variability, arable land per capita, and off-farm economic opportunities. By comparison, the combined determining force of factor pairs was stronger than the single-factor determinants. In the last decade, it was proposed that environmental factors not only directly contributed to rural out-migration but also played an indirect role through the socioeconomic drivers [32,38]. Moreover, it was found to be noteworthy that rural population losses were not only affected in a combined manner through the interactions between environmental and socioeconomic factors but they were also affected by the interactions between the different natural environmental factors, location, and natural environmental factors, agricultural economic opportunities and nonagricultural economic opportunities.

In terms of environmental and economic factors, almost all the environmental factors were at work with all of the economic factors in population decline. In the period 2000–2010, their combined influence was mainly reflected by the annual average rainfall and economic factors, and annual average temperature and economic factors, and they also had coefficients of more than 20% from 2010–2017. The reasons were mainly that the inadequate, infertile land rarely generated enough revenue to attract people to stay in their hometown, while the poor agrometeorological conditions also enhanced the influence of farmland by reducing the harvest in the cultivated land, especially with inadequate rainfall, low accumulated temperature, and climatic instability. Moreover, as Yu et al. found, Chinese rural residents often did not migrate out to work when their hometowns provided off-farm jobs [54]. Thus, for the rural townships with inadequate and poor cropland or poor agrometeorological conditions, they would suffer from a more serious population decrease when they also failed to provide enough off-farm jobs. 

For the natural environment variables, the annual average temperature obtained a coefficient of more than 20% with all the other natural variables in the two periods (in Table 4). Our study area, the northern mountains of Hebei province, is located in the transition region from a warm temperate zone to a middle temperate zone, and also in the crisscross area of Inner Mongolia Plateau, Taihang mountains, and Yan mountains, and North China Plain. Accordingly, the local climatic condition for agricultural production which mainly included rainfall and heat varied a lot and was more vulnerable to climate change. Thus, the rural areas with inadequate rainfall would suffer from poorer agricultural yields and consequently more serious out-migration and population loss when there were not good thermal conditions and an unstable climate. Moreover, in that case, the mountain rural areas often suffered from a greater population loss, as people there could not maintain their livelihoods by farming and animal husbandry activities in the condition of scarce arable land and adverse agricultural climatic conditions. This indicated that multiple adverse agricultural climate conditions often exacerbated rural migration. It is worth mentioning that the natural factors’ interactive effect may be different in other geographic regions.

As Table 3 shows, the natural environmental factors affected rural depopulation a lot through the combined effect with the location. The reasons were probably that the rural townships with inadequate rain-thermal conditions and climate disaster risk, especially inconsistent rainfall, drought, frost, and hailstone, restricted the development of agriculture and animal husbandry. Moreover, the rural townships far from the urban built-up area of the counties and prefecture-level cities also suffered from the poor access to public services, which would lead to out-migration and population loss. All these disadvantages combined exacerbated population decline. In our study area, Bashang Plateau and the Yanshan mountains in northern Hebei province were not only far away from urban areas but also had unfavorable and unstable agricultural climate conditions. Thus, there had been a dramatic population decline.

As Table 4 shows, the natural environmental factors and social factors combined also greatly affected rural depopulation. Especially for the population density, it had a coefficient of more than 46% with all the natural environmental factors. The spatial pattern of rural population decrease in northern Hebei Province strongly supports this finding. The most dramatic population decline often happened in the Bashang plateau, Yan mountains, and the transitional area from Bashang plateau to Hebei Piedmont, where there was scarce cropland, unfavorable agricultural climatic conditions, or high sensitivity to climate change. The low population density would enhance the influence of these environmental factors on rural depopulation by the inadequate and unsustainable provision of public services.

### 4.7. Dynamics of Local Drivers of Rural Depopulation

Comparing comprehensively the periods 2000–2010 and 2010–2017, the main drivers of rural population decline changed significantly. As shown in Table 3, during the years 2000–2010, rural depopulation was mainly driven by arable land per capita (rank 2nd) and natural environmental variables, i.e., annual average temperature (rank 1st), annual average rainfall (rank 3rd), and rainfall variability (rank 5th). Moreover, as shown in Table 4, the above-mentioned agricultural meteorological factors and per capita arable land also played a major role in the interactive effects. However, in 2010–2017, the main drivers of rural depopulation were concentrated on the social and economic dimensions. Specifically, rural depopulation was mainly driven by population density (rank 1st), off-farm economic opportunities (rank 2nd), agricultural economic density (3rd), and the fertility of cultivated land (rank 5th). The location’s effect also mattered, with the rank of location variables rising significantly (see Table 3). Additionally, the above-mentioned factors also played a leading role in the interaction process (in Table 4), especially population density and off-farm economic opportunities. Overall, the main drivers of rural population decline from 2000 to 2010 were closely related to agriculture. While the main drivers from 2000 to 2017 which altered population density were non-agricultural economic factors and location. 

The change in the main driver for rural depopulation could be explained by the following two aspects. On one hand, rural livelihood had become more diversified in the last decades [61]. The rural residents were increasingly dependent on nonagricultural sectors, not agriculture, to survive. Especially for the mountain areas in northern Hebei Province, the Program of Converting Farmland to Forest reduced the cropland resources of peasant households, and, consequently, lead more people to migrate out to participate in non-agricultural employment. Hence, from 2000–2010, rural depopulation was more sensitive to the natural environment variables and arable land per capita which determined agricultural yields. While during 2010–2017, it was less dependent on agroclimatic conditions and cropland, and more dependent on off-farm economic opportunities and location, which mattered with non-agricultural industry development and accessibility of public services. Additionally, in the years 2010–2017, with the support of policies for rural poverty alleviation, many rural areas made remarkable achievements in the development of agricultural-sideline food processing industries (such as wine, food production, and meat-packing), facility agriculture (such as vegetable, fruit, and edible mushrooms), breeding industry, tourism, and so on. The progress in these sectors had attracted many laborers to work locally. On the other hand, rural population decline often was a self-reinforcing process [23]. As a result, the rural depopulation from 2010 to 2017 was also largely influenced by the population density in 2010, which was determined by rural depopulation from 2000 to 2010.

## 5. Conclusions and Implications

A better understanding of rural depopulation is essential for designing policies to revitalize rural areas. This paper took the mountains of northern Hebei province as a case study area, to analyze the spatial pattern and local influential factors of rural depopulation with a new spatial statical approach, i.e., GeoDetector. It contributes to the existing literature by providing new accurate empirical evidence about local drivers of rural population decline in less developed mountainous areas, which reveals both the local effective drivers of rural depopulation and the combined effect of multi-drivers on rural depopulation. We also revealed the dynamics of the push of rural depopulation on time series.

Our study shows that most rural townships in the study area experienced a population decline from 2000 to 2017. However, under the influence of rural poverty alleviation projects, compared to the period 2000–2010, the population growth areas significantly expanded during 2010–2017, while the sharp depopulation areas and severe depopulation areas experienced shrinkage. The rural depopulation varies greatly across the rural townships in the mountains of northern Hebei province and demonstrates striking spatial correlation while revealing the distribution of clusters. Bashang Plateau in northwest Zhangjiakou and the mountains in northern Chengde and western Zhangjiakou suffered sharp and remarkable rural depopulation, forming serval zones of serious rural depopulation. While in most rural townships in the areas along Beijing and central-south Chengde, a relatively mild and slightly decreasing rural population was noted, with several zones of slightly rural depopulation forming there. The pattern of rural depopulation is in accordance with the terrain. Rural depopulation tends to be intensive in plateau and mountainous areas, while relatively milder in the intermontane basin, the hill areas at medium or low attitudes and the piedmont plain. This study also concludes that rural depopulation often occurs in remote rural areas far away from regional central cities. In addition, we also found that the rural area adjacent to the capital towns of counties often experienced a strong decline in the rural population. 

Rural depopulation under the influence of urbanization is also driven by multiple local drivers, including the natural environment, economic opportunities, location, and social and demographic factors. The local influential factors identified by GeoDetector conclude that agricultural suitability of natural environment and climate changes is the trigger of rural depopulation, but the influence effect tends to weaken. Economic opportunities, which are indicated by arable land per capita, fertility of cultivated land, agricultural economic density, and off-farm economic opportunity, played a key and enhancing role in rural depopulation. The sparse population density and inadequate public services strengthen the rural depopulation. The location and distances to major central towns and municipal districts, contribute to the rural depopulation and have an enhanced effect. Additionally, these factors also drive rural depopulation through interaction with each other, and the effect of paired interaction is stronger than that of a single factor. It is found to be noteworthy that rural population losses are not only affected in a combined manner through the interactions between environmental and socioeconomic factors but also affected by the interactions between different natural environmental factors, location, and natural environmental factors, agricultural economic opportunities, and nonagricultural economic opportunities. Furthermore, the driving force of rural population decline is dynamic. The rural depopulation from 2000 to 2010 is mainly driven by factors concerning agricultural production. While the main drivers from 2000 to 2017 are attributable to population density, non-agricultural economic factors, and location. 

Our results further the conclusion that rural depopulation is a complex, multidimensional process where natural environmental and socio-economic factors, different natural environmental factors, location and environmental factors, and agricultural economic opportunities and nonagricultural economic opportunities are interlinked. 

In comparison, this paper systematically reveals the local factors associated with rural population change based on a quantitative method and the interaction effects between multiple factors. The results potentially provide empirical evidence for a deeper and clearer understanding of rural depopulation. The results may also inspire that, in addition to the macro pull associated with urbanization, we should increase attention to the impacts of local factors and the spatial correlations on rural depopulation. Additionally, this study also enlightens us that GeoDetector, which is good at dealing with different qualitative and quantitative variables and identifying the interactions of multiple factors on dependent variables, is well suited for application in the field of population geography, especially in areas with heterogeneous geographical environments.

Meanwhile, this study also provides significant references for China’s rural revitalization and sustainable development. The pattern of rural population change enlightens us that under the general trend of rural population loss, some rural areas still have the potential and possibility of revitalization. To avoid rural recession resulting from depopulation, it is important to adjust and optimize the rural settlement system in a timely way according to the dynamic of local population densities, natural environments for farming, cultivated land resources, and nonagricultural industries. Moreover, in the implementation of a rural revitalization strategy, we should attach great importance to the role of industrial revitalization in alleviating population loss and rural recession. The spatial allocations of public services and featured industries, such as fruit planting industries, processing industries for agricultural products, and rural tourism, could be used to guide the redistribution of the rural population.

Finally, there are still some open issues regarding rural depopulation left for further research. First, the method of using GeoDetector is appropriate for identifying the factors associated with rural depopulation and their interaction effects, but it is of little utility to examine the positive or negative relationship between explanatory variables and rural depopulation. Furthermore, it does not allow us to integrate potential variables from external macro and local-level. Further studies employing multilevel models or spatial econometric models to investigate the drivers of population change will add more accurate and clearer empirical evidence. Second, it is expected that a better understanding of rural depopulation based on both in-depth surveys and quantitative studies can be attained. Moreover, it is worth considering more potential factors associated with the change in rural population, such as policies that are not considered in our study owing to the unavailability of data.

## Figures and Tables

**Table 1:** Information about local influential factors of rural depopulation.

Variables	Description of the Variables	Data Source
**Natural environment**		
X1 Annual average rainfall	The average annual precipitation from 1980 to 2010 (mm).	The monthly data set of China’s monthly surface climatic data values on the National Meteorological Science Data Sharing Service Platform. The range of weather stations includes Mongolia, Shanxi, Hebei, Beijing, and Tianjin areas.
X2 Annual average temperature	The annual average temperature between 1980 and 2010 (0.1 °C).	
X3 Rainfall variability	The sum of the squares of deviations of the precipitation during the period ranging from 2000 to 2009, in which the annual average precipitation took the mean precipitation from 1980 to 2010 (mm^2^ × 10^4^).	
X4 Terrain	It is denoted by the geomorphic types. The geomorphic types in the study area can be divided into five categories, including plains at middle or low altitudes, low-relief mountains, and hills at low altitudes; medium-relief mountains at middle altitudes, loess ridges, and hills at middle altitudes, hills, and high-relief mountains at medium or high altitudes.	The data were obtained from the Resource and Environmental Science Data Centre of the Chinese Academy of Sciences (Data Center of Resources and Environmental Sciences, Chinese Academy of Sciences; http://www.resdc.cn/ (accessed on 20 March 2017).
**Rural economic opportunities**		
X5 Arable land per capita	It is calculated by dividing the total cultivated area by the total population.	Hebei Rural Statistical Yearbook
X6 Fertility of cultivated land	The average yield per unit area of cultivated land during the years ranging from 2000 to 2004 was adopted to estimate the land fertility (ton/hectare).	Hebei Rural Statistical Yearbook
X7 Agricultural economic density	The total output value of the agricultural, forestry, animal husbandry, and fishery in per unit administrative area (10,000 yuan/km^2^).	Hebei Rural Statistical Yearbook
X8 Off-farm economic opportunity	Total business income of rural township enterprises (10,000 yuan).	Hebei Rural Statistical Yearbook
X9 Rural residents’ income	The per capita net income of rural residents (yuan).	Same data source as the above
**Location factors**		
X10 Distance to county-level capital	Based on road network data, the path distances (km) from the townships to prefectural capitals and county-level capitals were extracted by the cost-distance analysis tool in ArcGIS platform.	The vector road network data was provided by the local planning bureau.
X11 Distance to the prefectural capital		
**Social demographic factors**		
X12 Population density	Total population/administrative area (persons/square kilometer).	Township data set of 2000 Census
X13 Provision of public services	It can be represented by the townships’ fiscal revenue (10,000 yuan).	Hebei Rural Statistical Yearbook

Note: The destimulant variables include X1, X2, X5–X9, and X10–X13. The stimulant variables include X3, X10, and X11. The type of X4 terrain is uncertain, and it’s depending on the specific types of landforms.

**Table 2:** Descriptive statistics of rural population change in the study area.

Statistical Item	Min	Max	Mean	Standard Deviation	Number of Rural-Township
Population size of 2000 (person)	1265	51,399	14,126	7880	442
Population size of 2010 (person)	1204	51,877	13,124	8082	442
Population size of 2017 (person)	1077	71,129	12,847	8874	442
Size of population change (2000–2010) (person)	−17,519	26,080	−1002	2338	442
Size of population change (2010–2017) (person)	−8854	31,372	−272	3122	442
Rate of population change (2000–2010) (%)	−66.30	190.69	−8.69	15.50	442
Rate of population change (2010–2017) (%)	−62.86	230.76	−3.06	24.05	442
Size of rural depopulation (2000–2010) (person)	11	17519	1571	1604	356
Size of rural depopulation (2010–2017) (person)	5	8854	1652	1593	277
Rate of rural depopulation (2000–2010) (%)	0.07	66.30	12.83	9.99	356
Rate of rural depopulation (2010–2017) (%)	0.03	62.86	14.39	12.71	277

**Table 3:** Results of factor detection for the rural depopulation rates.

Explanatory Variables		2000–2010		2010–2017	
		*q*	Rank	*q*	Rank
Natural environmental factors	X1 Annual average rainfall	0.1334 ***	3	0.0185	-
	X2 Annual average temperature	0.2143 ***	1	0.1692 ***	4
	X3 Rainfall variability	0.1143 ***	5	0.0264	-
	X4 Terrain	0.0708 ***	7	0.1009 ***	10
Economic factors	X5 Arable land per capita	0.2038 ***	2	0.1041 ***	9
	X6 Fertility of cultivated land	0.0351 ***	11	0.1363 ***	5
	X7 Agricultural economic density	0.1219 ***	4	0.2235 ***	3
	X8 Off-farm economic opportunity	0.0663 ***	8	0.2528 ***	2
	X9 Rural residents’ income	0.0451 ***	9	0.1206 ***	8
Location factors	X10 Distance to county-level capital	0.0068	-	0.1238 ***	7
	X11 Distance to prefectural capital	0.0266 **	12	0.1266 ***	6
Social factors	X12 Population density	0.0837 ***	6	0.4427 ***	1
	X13 Provision of public services	0.0383 ***	10	0.0967 **	11

Note: ***, ** represent the significance levels of 1%, 5%, respectively.

**Table 4:** The coefficients of interaction detector between various factors on rural depopulation during 2000–2010 and 2010–2017 (%).

	Variables	X1	X2	X3	X4	X5	X6	X7	X8	X9	X10	X11	X12	X13
2000–2010	X1 Annual average rainfall	13.34												
	X2 Annual average temperature	27.31	21.40											
	X3 Rainfall variability	16.29	26.08	11.43										
	X4 Terrain	18.71	25.72	20.05	7.08									
	X5 Arable land per capita	25.79	27.51	21.64	27.02	20.38								
	X6 Fertility of arable land	17.77	24.26	14.31	10.86	23.23	3.51							
	X7 Agricultural economic density	22.82	24.97	19.60	22.22	26.09	13.45	7.46						
	X8 Off-farm economic opportunities	27.01	27.95	25.12	18.19	25.94	14.63	14.51	6.63					
	X9 Rural residents’ income	23.56	26.09	21.12	18.27	24.61	10.53	12.92	10.97	4.51				
	X10 Distance to county-level capitals	20.75	24.33	20.11	14.93	26.10	7.36	9.81	8.86	9.89	0.69			
	X11 Distance to prefectural capitals	19.10	27.33	19.06	16.05	30.07	11.34	10.71	11.12	9.60	11.15	2.66		
	X12 Population density	26.24	25.12	24.70	21.14	27.80	17.18	12.61	12.28	12.84	10.09	10.55	8.37	
	X13 Provision of public services	22.88	24.15	19.52	14.26	25.32	13.12	11.80	9.49	8.95	6.15	9.55	12.58	3.83
2010–2017	X1 Annual average rainfall	1.85												
	X2 Annual average temperature	22.65	16.92											
	X3 Rainfall variability	10.58	23.32	2.64										
	X4 Terrain	17.14	25.99	14.13	10.09									
	X5 Arable land per capita	17.42	19.99	16.82	25.77	10.41								
	X6 Fertility of cultivated land	18.98	26.36	20	24.29	17.76	13.63							
	X7 Agricultural economic density	29.81	33.13	24.44	25.27	30.98	29.43	22.35						
	X8 Off-farm economic opportunity	34.46	36.44	29.45	34.03	30.73	34.07	37.26	25.28					
	X9 Rural residents’ income	21.59	27.96	14.83	19.27	22.32	24	28.22	32.1	12.06				
	X10 Distance to county-level capital	18.69	28.25	21.04	18.87	21.85	26.42	27.6	31.25	21.4	12.38			
	X11 Distance to prefectural capital	20.01	25.75	17.19	21.62	24.09	24.5	29.42	35.12	22.42	21.97	12.66		
	X12 Population density	48.57	46.42	46.62	46.75	45.93	49.29	48.33	51.39	48.55	46.68	46.1	44.27	
	X13 Provision of public services	16.88	24.09	13.3	22.25	17.63	22.83	32.72	29.68	18.18	19.68	20.15	46.65	9.67

Note: 
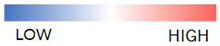
 From blue to red, the interaction detection coefficient increases gradually.

## Data Availability

Not applicable.
